# Volitional enhancement of firing synchrony and oscillation by neuronal operant conditioning: interaction with neurorehabilitation and brain-machine interface

**DOI:** 10.3389/fnsys.2014.00011

**Published:** 2014-02-06

**Authors:** Yoshio Sakurai, Kichan Song, Shota Tachibana, Susumu Takahashi

**Affiliations:** ^1^Department of Psychology, Graduate School of Letters, Kyoto UniversityKyoto, Japan; ^2^Laboratory of Neural Circuitry, Graduate School of Brain Science, Doshisha UniversityKizugawa, Japan

**Keywords:** operant conditioning, synchrony, oscillation, neurorehabilitation, brain-machine interface

## Abstract

In this review, we focus on neuronal operant conditioning in which increments in neuronal activities are directly rewarded without behaviors. We discuss the potential of this approach to elucidate neuronal plasticity for enhancing specific brain functions and its interaction with the progress in neurorehabilitation and brain-machine interfaces. The key to-be-conditioned activities that this paper emphasizes are synchronous and oscillatory firings of multiple neurons that reflect activities of cell assemblies. First, we introduce certain well-known studies on neuronal operant conditioning in which conditioned enhancements of neuronal firing were reported in animals and humans. These studies demonstrated the feasibility of volitional control over neuronal activity. Second, we refer to the recent studies on operant conditioning of synchrony and oscillation of neuronal activities. In particular, we introduce a recent study showing volitional enhancement of oscillatory activity in monkey motor cortex and our study showing selective enhancement of firing synchrony of neighboring neurons in rat hippocampus. Third, we discuss the reasons for emphasizing firing synchrony and oscillation in neuronal operant conditioning, the main reason being that they reflect the activities of cell assemblies, which have been suggested to be basic neuronal codes representing information in the brain. Finally, we discuss the interaction of neuronal operant conditioning with neurorehabilitation and brain-machine interface (BMI). We argue that synchrony and oscillation of neuronal firing are the key activities required for developing both reliable neurorehabilitation and high-performance BMI. Further, we conclude that research of neuronal operant conditioning, neurorehabilitation, BMI, and system neuroscience will produce findings applicable to these interrelated fields, and neuronal synchrony and oscillation can be a common important bridge among all of them.

## Operant conditioning of neuronal firing

**Figure 1:**
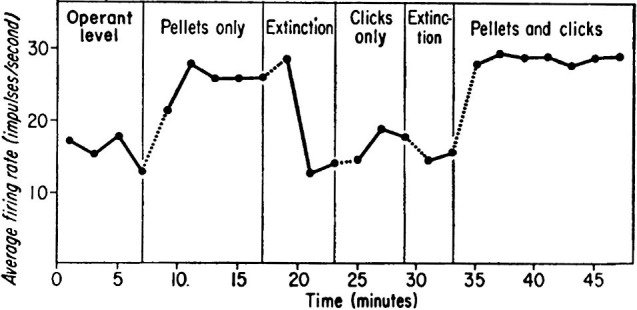
**Data showing firing rate of a motor cortex neuron of the monkey as a function of reinforcement schedule of neuronal operant conditioning**. During operant level and extinction periods neither food nor click feedback was presented. During “Pellets only” period, the highest firing rates were reinforced with delivery of a food pellet without click feedback. During “Click only” period, a click was presented for each firing of the neuron; finally, both pellets and clicks were provided. (From Fetz, 1969, with permission).

When we require learning of volitional enhancement of a certain behavior, operant conditioning (Skinner, 1974; Reynolds, 1975) should be the first choice. The voluntary behavior immediately followed by reward, i.e., having contingency of reward, soon becomes more frequent, and humans and animals volitionally conduct the behavior more frequently to get more reward. Based on such methodology, an intriguing method of learning of volitional enhancement in neuronal firing has been developed and called neuronal operant conditioning, in which rewards are given for modulations of neuronal firing which are not linked to overt behaviors. Since Olds (1965) and Fetz (1969) published their pioneering research, conditioned enhancement of neuronal firing has been frequently reported in animals and humans. In particular, Fetz and collaborators (Fetz, 1969; Fetz and Finocchio, 1971; Fetz and Baker, 1973) had established the methodology of neuronal operant conditioning and reported that monkeys could control firing rates of individual neurons in the motor cortex (Figure 1). Following these pioneering and memorable experiments, several intriguing studies by Fetz and other researchers have been published.

Recently, for example, Kobayashi et al. (2010) has demonstrated a remarkable capacity of single neurons to be driven by volition by adapting to specific operant requirements. This experiment set variable relationships between levels of single-neuron activity in the monkey prefrontal cortex and rewarding outcomes. Prefrontal neurons changed firing rates according to the specific requirements for gaining reward, without the monkeys making a motor response, and indicated that neuronal firings constituted a volitional operant response enhanced by reward. The control task of the experiment suggested that these changes of firing were unlikely to reflect simple reward predictions. In humans, Cerf et al. (2010) demonstrated that subjects can regulate firing rates of single neurons in the medial temporal lobe (MTL) to obtain the rewarding outcome that visual images they liked to see became clearer on the computer screen in front of them. The study recorded from single neurons in patients implanted with intracranial electrodes for clinical reasons. The subjects looked at a hybrid superposition of two images representing familiar individuals, landmarks, objects, or animals and had to enhance one image at the expense of the other, competing one. Simultaneously, the firing of MTL neurons was decoded in real time to control the content of the hybrid, i.e., making one of the superposed images clearer than the other. The subjects reliably regulated the firing rate of these neurons, increasing the rate of some while simultaneously decreasing the rate of others. The subjects achieved this by focusing onto one image, which gradually became clearer on the computer screen, thereby overriding sensory input. On the basis of the firing of these MTL neurons, visual images in the subject’s mind were visualized on an external display, which functioned as reward.

The most recent progress is reported by Arduin et al. (2013). They employed a strategy of accessing reward by controlling a prosthetic device with self-generated neuronal firing from a single neuron. They recorded multiple neurons from motor cortical areas in rats for controlling a linear actuator with a water bottle. To receive reward of water, the rats had to move the bottle until it reached a zone for drinking by raising and maintaining firing rate of each neuron above a high threshold. They defined the time the bottle took to reach the drinking zone after trial onset as time-to-reward. If the time-to-reward distribution during trials significantly differed from that during waiting periods, the single neuron was considered an operantly conditioned neuron (Opris et al., 2011b).

**Figure 2:**
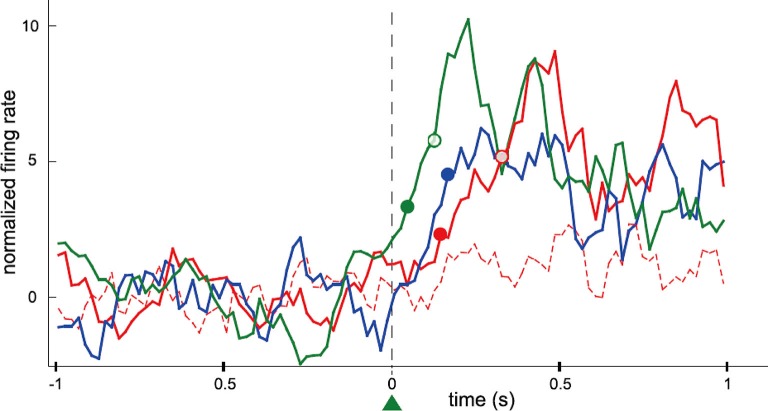
**Data showing differences in the rank of activation between conditioned neurons and simultaneously recorded neighboring neurons**. Firing rate of motor cortex neurons in the rat was operantly conditioned. This presents perievent time histogram of neuronal activity normalized and centered on trial onset, for four neurons simultaneously recorded during the same session (green: the conditioned neuron; blue: a previously conditioned neuron; red: two neighboring neurons never conditioned). Filled and empty circles of different colors represent the latency of the neurons until their firings exceeded the thresholds at 2 and 5 standard deviations (SD), respectively. One of the neurons (red dashed line) did not have a measurable latency for that recording session. The latency of the blue neuron could only be defined for the 2 SD threshold. Additional (*n* = 20) neurons that were simultaneously recorded during that recording session have not been included for sake of clarity. Bin size: 20 ms; each value is the *z*-transform of the firing rate integrated over a sliding window of 100 ms. Latencies were calculated using a 20 ms bin scale. (From Arduin et al., 2013, with permission).

The firing rates of conditioned neurons increased instantaneously after a trial onset and the bottle entered the drinking zone within a very short time. The time-to-reward for the conditioned neurons soon decreased and exhibited significant difference compared to that for non-conditioned neurons. The majority of the conditioned neurons increased firing rates reliably and instantaneously after trial onset despite the absence of any temporal requisition. Furthermore, the conditioned neurons fired more frequently, instantaneously, and strongly than the neighboring neurons that were simultaneously recorded around the conditioned neurons (Figure 2). The authors concluded that only the operant-conditioned neurons possessing significantly increased firing rates take the lead as “master neurons”, that exhibit most prominent volitionally driven modulations in a small neural network.

Such on-going progress of research into neuronal operant conditioning confirms the possibility of volitional enhancement of activity for specific individual neurons. However, possibility of chance reinforcement of a body movement rather than neuronal activity should always be checked. The question is whether operantly conditioned neuronal firing is directly controlled in certain central pathways or through an accidentally reinforced body movement which generates activity in the whole pathways leading to the muscles, including corollary discharge and proprioceptive and sensory feedbacks. Concerning involvement of the proprioceptive feedback, Wyler et al. (1979) reported that section of pyramidal tract and ventral rhizotomies disrupted operant conditioning of firing of precentral neurons and suggested that the precentral neurons were operantly controlled through the proprioceptive feedback from the peripheral mechanoreceptors. However, such lesions of nerve fibers could yield neuronal death and/or reorganization of neuronal networks, which may disrupt normal neuronal activity and/or potential for learning and conditioning. Intact brains and input-output pathways should be employed to answer the question of central pathways vs. body movements. Using the intact brains and pathways, several former and recent studies (Fetz and Finocchio, 1971; Koralek et al., 2012; Engelhard et al., 2013; Sakurai and Takahashi, 2013) reported the absence of specific body movements or muscle activity during the operant conditioning of neuronal firing. Although precise and detailed mechanisms that make neuronal operant conditioning possible are not clear yet, it is apparent that neuronal activity can be operantly conditioned without body movement and enhanced volitionally by setting direct contingency between changes of neuronal activity and delivery of reward.

## Operant conditioning of firing synchrony and oscillation

The previous studies surely have confirmed robustness of operant conditioning of neuronal activity. Most of them, however, had a bias due to an exclusive focus on the firing rates of individual neurons of neocortices. Neuronal operant conditioning should be used to explore the extent to which synchronous activity in neurons can be volitionally enhanced. Synchronous neuronal activity reflects functional connectivity among multiple neurons and had not been the target of neuronal operant conditioning, though the brain functions can be considered to be realized by activities not of individual neurons but of ensembles of populations of neurons interrelated with each other. Therefore, enhancement of neuronal activity related to brain functions could be realized more reliably by operant conditioning of such ensemble activity of neuronal populations typically reflected by synchronized firing of multiple neurons.

**Figure 3:**
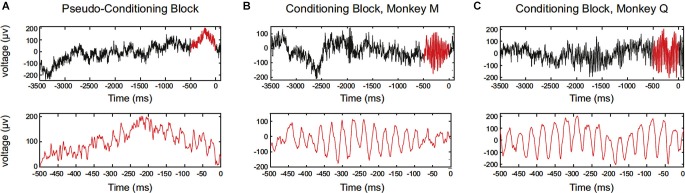
**Data showing single-trial examples of LFP aligned to reward delivery (0)**. Oscillatory firing of motor cortical neurons of the monkey was operantly conditioned. **(A)** Data for monkey M (session #20). Upper panel shows a raw LFP trace in the pseudoconditioning block. The 500 ms period before reward is highlighted in red. Lower panel is an expanded view of the 500 ms period before reward. **(B)** The same as in **(A)**, but for a trial in the conditioning block. **(C)** The same as in **(A)**, but for a trial in the conditioning block, monkey Q (session #17). (From Engelhard et al., 2013, with permission).

Engelhard et al. (2013) has recently reported that periodically synchronized activity, i.e., oscillatory activity, of motor cortical neurons can be enhanced by operant conditioning. The study has succeeded to train monkeys to increase motor cortex low-gamma waves of local field potential (LFP) (Figure 3). Single-neuron firing was recorded, and the enhancement of operantly conditioned oscillatory waves was accompanied by a correlated increase in the synchrony of the entrained neurons. This relation of LFP and neuronal firing can be explained by the fact that LFPs are produced by postsynaptic potentials, and periodicity in neuronal firing would be associated with periodicity in LFPs. They also documented the spatial extent of neurons entrained with the operantly conditioned oscillatory activity. Over the extent of 4 × 4 mm electrode grids, enhanced gamma power in the LFP and phase locking of neuronal firings occurred in a broad range (approximately 500 μm), and depth of entrained modulation decreased as a function of distance from the operant conditioning sites of electrodes. The study also confirmed that the enhancement of oscillatory activity was not associated with any observed movements or increases in muscle activity. From these findings, the authors argue that the findings link volitional control of LFP oscillations and neuronal-firing synchrony.

The low-gamma oscillations have been found in many different brain areas and are considered to be associated with different functions such as attention, perception, cognition, and computation (Herrmann et al., 2010) and to play as neural synchrony both within (Salinas and Sejnowski, 2001) and between (Siegel et al., 2012) brain areas. Therefore, the results of Engelhard et al. (2013) are ground breaking, whereby monkeys demonstrated the ability to directly modulate and enhance specific patterns of synchrony of many neurons in somewhat broad ranges, which may be related to several motor functions of the brain.

On the other hand, it is desirable to directly demonstrate operant enhancement of firing synchrony among individual neurons located closely in restricted smaller ranges. For such experiments, precise separation of extracellular firing from closely neighboring neurons in real time is required. It had been difficult, however, for traditional spike-sorting techniques (Lewicki, 1994; Fee et al., 1996), primarily because spike waveforms overlap on a common electrode when nearby neurons fire coincidently. To address this problem, we (Takahashi et al., 2003a,b) developed a unique method of spike-sorting using independent component analysis (ICA; Comon, 1994) with a specific multielectrode (Takahashi and Sakurai, 2005, 2007, 2009a,b). The method allows sorting of the firings of closely neighboring neurons in real time and the detection of firing synchrony. Using this technique, we have recently reported that synchronized firing of closely neighboring neurons in rat hippocampus can be enhanced by neuronal operant conditioning (Sakurai and Takahashi, 2013).

**Figure 4:**
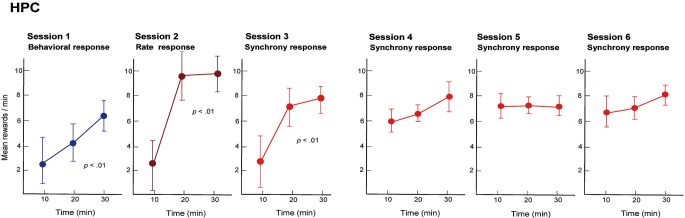
**Mean quantities of pellets, reward in the operant conditioning, delivered by behavior (session 1), firing rate above preset criteria (session 2) and firing synchrony above preset criteria (sessions 3–6) during each 30 min conditioning session**. Neuronal firing rate and synchrony were obtained from a group of neighboring neurons in the hippocampal CA1 of the rat and operantly conditioned. In sessions 1–3, means were calculated for three periods (10–20, 21–30 and 31–40 min) of each session. Data recorded for the first 10 min of each session are not presented because the first 10 min were used to perform behavioral shaping (session 1) and selection of criteria for the neuronal activities (sessions 2 and 3). In sessions 4–6, means were calculated for periods of 0–10, 11–20 and 21–30 min of each session because criterion selection had been conducted in session 3 and was thus unnecessary. (From Sakurai and Takahashi, 2013, with permission).

**Figure 5:**
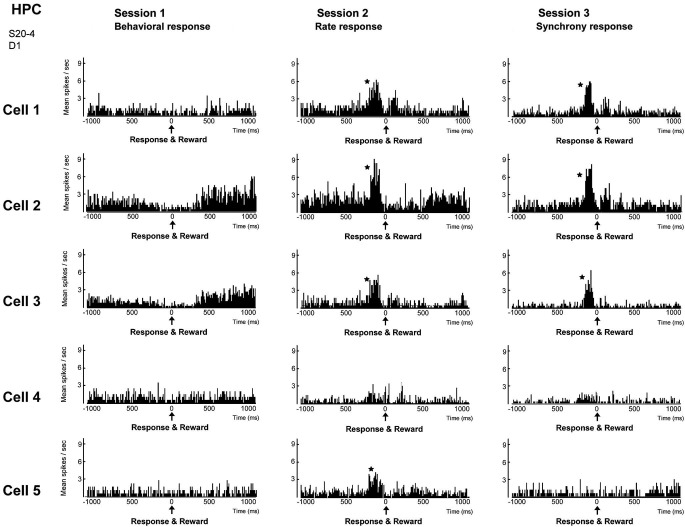
**Firing-rate histograms of five individual neurons comprising a group of neighboring neurons in the hippocampal CA1 in sessions 1–3**. The center (0) of each histogram means the time when behavior (session 1), firing rate above preset criteria (session 2) and firing synchrony above preset criteria (session 3) immediately followed by reward delivery were conducted. A bin is 10 ms and an asterisk indicates a significant increment of firing (confidence limit, *p* < 0.005). (From Sakurai and Takahashi, 2013, with permission).

**Figure 6:**
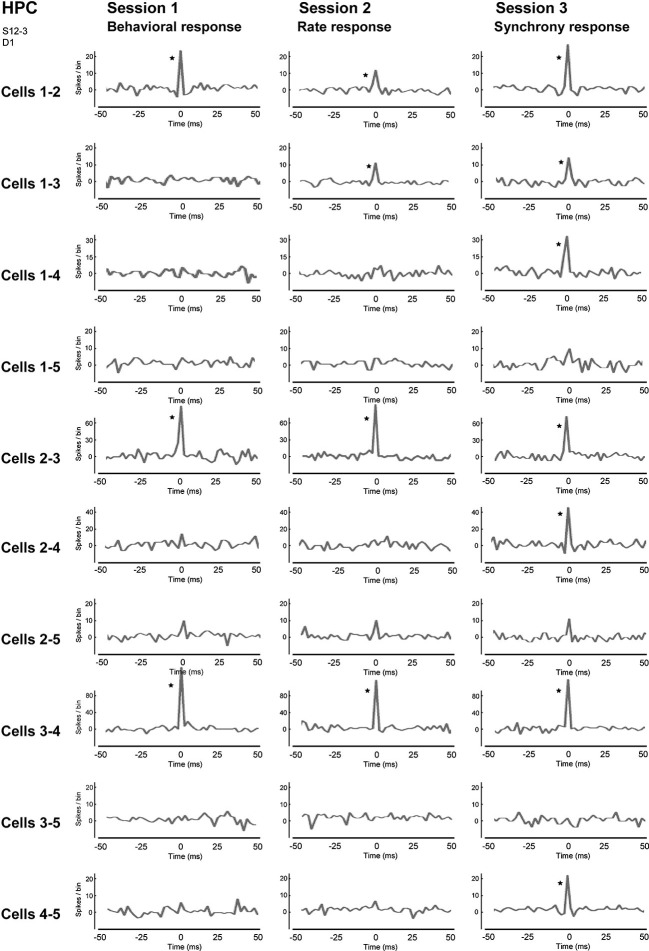
**Correlograms of all neuron pairs of five neighboring neurons in the hippocampal CA1 in sessions 1–3**. The center (0) of each correlogram means the time when two neurons fired simultaneously, i.e., achieved firing synchrony. A bin is 2 ms and an asterisk indicates a significant increment in firing synchrony (confidence limit, *p* < 0.005). (From Sakurai and Takahashi, 2013, with permission).

We trained rats to engage in a free-operant task in which nose-poke behavior was rewarded in session 1, and firing rates and synchrony of multiple neighboring neurons above preset criteria were rewarded in sessions 2 and 3, respectively. Placing contingency of reward on firing synchrony in session 3 resulted in selective enhancement of firing synchrony of the hippocampal neurons (Figure 4). Control experiments revealed that the enhancement of firing synchrony was not attributable to increments of behaviors or excitation caused by reward delivery. Analysis of the firing rates and synchrony of individual neurons and neuron pairs during the conditioning revealed that the firing rates and synchrony of some but not all neurons and neuron pairs increased in each group of neighboring neurons (Figures 5, 6). No firing enhancement was observed in any neurons and neuron pairs recorded by closely placed electrodes not used for the conditioning. From all these findings, we conclude that neuronal operant conditioning can lead to volitional enhancement of firing synchrony in a small group of neurons in a small restricted area in the hippocampus.

In that study, operant conditioning of firing synchrony was obtained in the hippocampus but not in the motor cortex. One explanation might be that the hippocampus has the high level of plasticity causing learning-related changes of firing synchrony among the neurons (e.g., Sakurai, 1996a, 2002). This explanation, however, does not exclude the possibility of motor cortical neurons to be conditioned in their firing synchrony. The hippocampal synchrony functions could be revealed at small timescales such as the bin (2–4 ms) used for the operant conditioning in our study (Sakurai and Takahashi, 2013), whereas in the motor cortex synchrony could be best functional at longer timescales such as that of low gamma oscillations. This assumption is apparently supported by the result of Engelhard et al. (2013) introduced above.

It should be noted that, as Fetz (2013) suggested, synchronous neuronal firing was detected not only as lasting and periodic, e.g., oscillations, but also as temporal and episodic. For example, Riehle et al. (1997) has reported that such temporally short and episodic synchrony of firing of motor cortical neurons can be detected during some specific behavior. Such synchrony was termed “unitary event” which appeared consistently at particular times in relation to an expected cue at times unrelated to sensory or motor events. Such episodic synchrony of firing should be a target of neuronal operant conditioning. Schmied et al. (1993) reported that humans could be operantly conditioned to increase some episodic synchrony of groups of motor cortical neurons. However, because synchronized firings can be caused by common synaptic inputs, such demonstration may be essentially equivalent to demonstrating enhancement of firing of the common input neurons. In contrast, periodic synchrony of firing represents a rhythmic phenomenon involving a different mechanism generating more prolonged circuit resonance (Fetz, 2013).

## Why synchrony and oscillation?—view on “cell assembly”

As described above, operant conditioning of oscillation and synchrony of multiple neurons can be indispensable to enhancing brain functions because they are realized by ensemble activities of populations of neurons that are functionally connected with each other. Such a functional population of neurons has been proposed to be “cell assembly” (Hebb, 1949), postulated to act as a functional unit that represents information in the working brain and underlie perception, learning, and memory for adaptive behavior (Eichenbaum, 1993; Sakurai, 1996b, 1999; Harris, 2005; Opris et al., 2013). The original concept of cell assembly was a theoretical notion and it could have value and be substantial when it accounts for experimentally observed phenomena. The experimental observations showing major properties of the cell assembly are, as Sakurai (1999) suggested, the task-related functional overlapping of individual neurons (Sakurai, 1994) and the task-dependent dynamics of the functional connectivity among the neurons (Sakurai, 1993). In particular, the latter phenomena, reflected as dynamically changing synchrony of firing of multiple neurons, has often been reported and regarded as the popular operational definition or indirect evidence of the activity of cell assemblies (Sakurai, 1996a, 2002; Riehle et al., 1997; Engel et al., 2001). Therefore, the target activity of neuronal operant conditioning should include not only firing rates but also firing synchrony of multiple neurons, as reported in Engelhard et al. (2013) and Sakurai and Takahashi (2013).

However, operant conditioning of the activity of cell assemblies is not an easy task because the ranges in the patterns of activation of cell assemblies, i.e., sizes of cell assemblies, have been postulated to be diverse (Sakurai et al., 2013). A cell assembly could be comprised of a small number of localized neurons or a large number of broadly distributed neurons (Eichenbaum, 1993). Therefore, neurons in the neocortices and the limbic structures, particularly the hippocampus, are expected to show various forms of firing synchrony, which represent dynamic and diverse representation by cell assemblies, in various behavioral tasks. Actually, several former studies have reported the task- and behavior-dependent dynamic synchrony of neurons in the wide ranges (Abeles et al., 1993; Vaadia et al., 1995; Seidemann et al., 1996; Engel et al., 2001; Opris et al., 2011a, 2012a,b) and the local ranges (Funahashi and Inoue, 2000; Constantinidis et al., 2001; Sakurai and Takahashi, 2008). We have reported task-dependent sharp synchrony of firing among the neighboring neurons, reflecting the small and localized cell assemblies, in the monkey prefrontal cortex, but at the same time, we have also found dynamically changing broad synchrony of firing among the distant neurons (Sakurai and Takahashi, 2006). The sizes of cell assemblies are certainly diverse and dependent on information representation and processing in behavioral tasks.

The diversity in the sizes of cell assemblies should be considered when neuronal operant conditioning is applied to enhance synchronized neuronal activity. Our study (Sakurai and Takahashi, 2013) has operantly enhanced firing synchrony of the small and localized groups of neighboring neurons, using the specific electrode and the spike sorting, in the rat hippocampus. Such synchronized firing among close neurons has been shown to be valid for some information processes. For example, Fujisawa et al. (2008) has reported clear synchrony of firing among neighboring neurons in the rat prefrontal cortex. The authors focused on the sharp peaks in cross-correlograms between pyramidal neurons and interneurons with millisecond time lags that were consistent with monosynaptic delays. The temporal relationships of the activities of neurons were examined during a working memory task. Numerous monosynaptic pairs between the pyramidal neurons and interneurons dynamically varied their peaks in the cross-correlograms across various phases of the task beyond the statistical accounting for the effects of covarying the firing rates of the neurons. This indicates that functional interplay among the close neurons linked by monosynaptic connections is working during the behavioral task. This finding was consistent with those of previous studies that have observed variance in the short-term synchrony between neuronal pairs as a function of behavioral performance and learning (Constantinidis et al., 2002; Baeg et al., 2007; Opris et al., 2011a, 2012a).

On the other hand, the study of Engelhard et al. (2013), introduced above, can be considered to have succeeded to operantly enhance activity of broader cell assemblies reflected by oscillatory low-gamma waves of LFP, because the oscillatory LFPs are produced by synchronized postsynaptic potentials of many neurons in broader ranges. Oscillatory activity in the motor cortex has been observed in many experiments and led various hypotheses about its possible functions, such as motor preparation and attention to aspects of movement (Murthy and Fetz, 1996; Donoghue et al., 1998). Oscillatory activity has also been documented most thoroughly in the visual system, where many experiments have suggested that the widespread periodicity is involved in top-down processing (Engel et al., 2001) and plays a role in long-range interactions between different cortical regions (Siegel et al., 2012).

Discussion is still ongoing about the actual functional role of oscillatory and synchronous activities. But with neuronal operant conditioning, as Fetz (2013) suggested, those activities become the independent variable in the experiments, and their effects on behavior are more compelling evidence of their functions. Actually, Keizer et al. (2010) has shown that volitionally increased gamma oscillation at occipital and frontal sites in humans surely improved performance on cognitive tests of sensory binding and memory. This result supports the notion that various information processes are generated by oscillatory activity in the motor and sensory cortices.

In addition to the findings of oscillation, synchrony of firings among individual neurons in broader ranges has been reported. Pipa and Munk (2011) trained monkeys to perform a short-term visual memory task and simultaneously recorded multineuronal activity from the prefrontal cortex with electrodes that were arranged in a square-shaped 4 × 4 grid with a distance between the nearest neighbors of 500 μm. The authors found firing synchrony of neurons with high temporal precision across the electrode sites. The frequency of synchrony was modulated depending on the behavioral performance and the specific stimuli that were presented. In particular, during the delay period, larger groups of up to 7 electrode sites showed performance-dependent modulation of the synchronous firings. These findings indicate dynamic activity of broad populations of distributed neurons that underlie the higher temporal organization of information being processed for the task performance.

Recent technological advances have made it possible to record from larger neuronal populations. New principal component analysis (PCA) methods (Peyrache et al., 2009; Lopes-dos-Santos et al., 2011) are suitable for detecting larger cell assemblies that are constructed from larger number of distributed neurons. However, classical methods, such as cross-correlation analyses, have merit in detection of detailed structures of functional connectivity between neighboring neurons. A combination of the new methods of PCA and the classical methods may be ideal in detecting diverse synchrony of neuronal activity and useful to selectively enhance activities of cell assemblies with different sizes.

## Relevance to neurorehabilitation and brain-machine interface

### Neurorehabilitation

Neuronal operant conditioning can elucidate the potential of neuronal plasticity (Dobkin, 2007) induced by conditioning of neuronal activity including synchronous and oscillatory activities. Such elucidation contributes to progress in the development of neurorehabilitation methods (Raskin, 2011), the majority of which attempt artificial enhancement of neuronal activity to compensate for loss of brain motor functions. A turning point of neuronal operant conditioning to be applicable may be the fact that it does not require selection of functionally specific neurons for the conditioning. It would not be possible to condition and enhance inherent motor neurons for compensation of motor functions because most motor-function losses are accompanied by loss of inherent motor neurons. Therefore, neuronal operant conditioning should have the potential to enhance any neuron and hopefully any brain region unrelated to the target functions to be compensated. This could be related to the theory of multipotentiality of the brain (John, 1980). That theory suggests that any neuron and region may contribute to the mediation of a diversity of functions and that many neurons and regions contribute to every function, but it does not imply that different neurons and regions are functionally equivalent or that different functions depends equally on diverse neurons and regions.

Actually in our study (Sakurai and Takahashi, 2013), the neurons showing rapid enhancement in firing rates and synchrony during the neuronal operant conditioning had been selected randomly and originally manifested no behavior-related activity responsible for motor responses. This finding indicates that neurons not initially involved in behavioral performance can be enhanced by the conditioning and subsequently utilized to compensate for loss of motor functions responsible for behavior. Such an indication had previously emerged from the findings of Moritz et al. (2008), who observed that monkeys could learn to use task-unrelated neurons to control an external device if they were provided with operant control training.

Besides the notion of non-selectivity of neurons, it is again noted that conditioning of oscillatory and synchronous activities are expected to lead to more effective neurorehabilitation. Synchronous oscillations in motor cortical neurons have been observed in many behavioral experiments, leading to hypotheses about its possible function. For example, it has been reported to occur during an instructed delay period prior to movement and then disappear during the overt movement, suggesting a role in motor preparation (Donoghue et al., 1998). Oscillations have also been observed to appear during a maintained precision grip (Baker et al., 1999) and free exploratory hand movements (Murthy and Fetz, 1996). It should be emphasized that these oscillations entrained both task-related and unrelated neurons equally, and coherent oscillations occurred over widespread cortical areas, including both hemispheres, but correlations between different cortical sites did not depend on the site’s relation to the task (Fetz, 2013). Consequently, inducing such oscillatory activity by operant conditioning could thus enhance several motor-related functions.

In addition to the motor-related functions, synchrony and oscillations are considered to be associated with attention, perception, cognition, and computation (Fries, 2009; Herrmann et al., 2010) and active both within (Salinas and Sejnowski, 2001) and between (Siegel et al., 2012; Terada et al., 2013) brain locations, as described previously. These indicate the possibility of enhancing such higher functions by conditioning of synchrony and oscillations of firing. An issue to be addressed is whether any neuron can be available for the conditioning to enhance the higher functions in the sensory and higher brain regions, as motor functions in the motor-related regions. Addressing this issue involves testing the validity of the view of multipotentiality of the brain (John, 1980) briefly introduced above.

### Brain-machine interface

**Figure 7:**
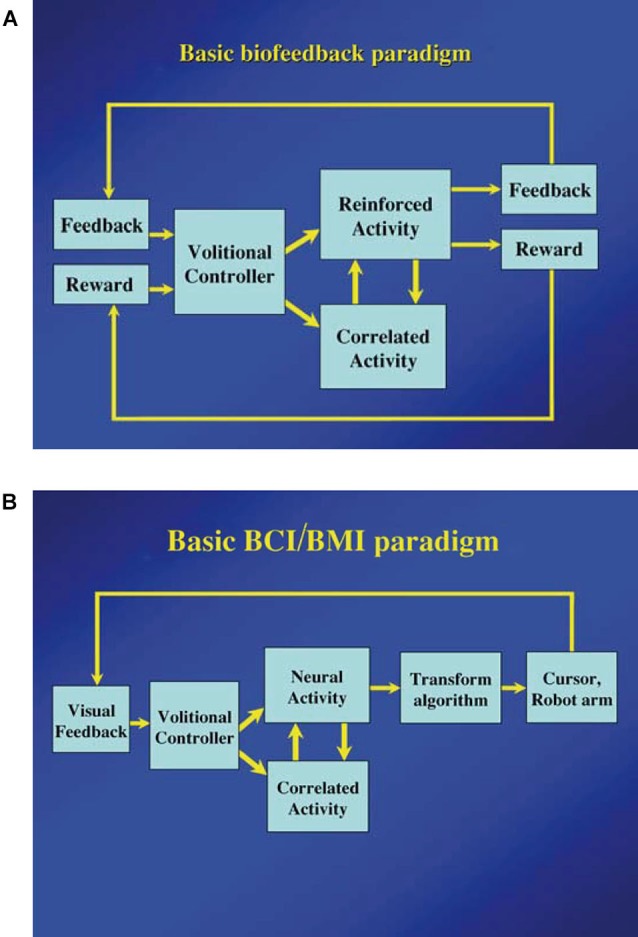
**(A)** Basic components of neuronal operant conditioning (biofeedback) paradigm. Feedback and reward are contingent on the reinforced activity and provided to the brain of the “volitional controller”. The correlated activity consists of additional neural or physiological activity either causally or adventitiously associated with the reinforced activity. (From Fetz, 2007, with permission). **(B)** Basic components of the brain–computer interface (BCI) or brain-machine interface (BMI) paradigms. Essential components are identical to those of the neuronal operant conditioning, except that feedback (usually visual) is provided by the controlled device or cursor, and a more sophisticated transform algorithm is typically used to convert neural activity to the requisite control signals. (From Fetz, 2007, with permission).

**Figure 8:**
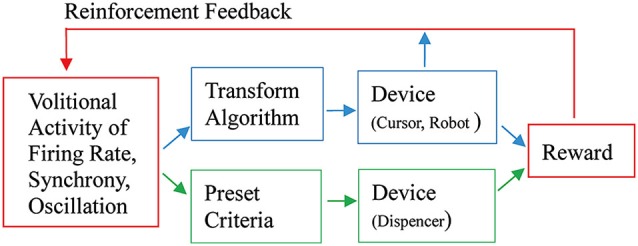
**An integrated view of basic components of neuronal operant conditioning (Figure 7A) and BMI (Figure 7B) paradigms.** The different stages are shown in blue and green parts, the former for BMI and the latter for neuronal operant conditioning, and the common stages are shown in red parts. See the text for detail.

As Fetz (2007) suggested, the basic paradigm for neuronal operant conditioning (neural biofeedback) is essentially identical to the paradigm for brain-machine interface (BMI) (Figure 7). BMI is for neuroprosthetic control of external devices by neuronal activity instead of behavior (Berger et al., 2008; Hatsopoulos and Donoghue, 2009; Nicolelis and Lebedev, 2009; Andersen et al., 2010; Green and Kalaska, 2011). Neuronal operant learning can elucidate the possibility of volitional control of neuronal activity and contribute to the development of BMI. One difference is the transform algorithm converting neural activity to the control signals operating the external device to get reward. Though this interposes an intermediate stage that may complicate the relationship between neural activity and device control, the final outcome is identical with that of neuronal operant conditioning, i.e., getting reward. The device control in BMI finally results in getting of reward and sometimes, particularly in humans, being able to control the device itself functions as reward. This leads to the conclusion that the basic strategy—volitional activity associated with getting reward is enhanced by reinforcement feedback—is identical between BMI and neuronal operant conditioning. Figure 8 summarizes the common and different stages in BMI and neuronal operant conditioning.

Although the development of invasive BMI is promising (Lebedev and Nicolelis, 2006), currently available BMIs are limited in terms of accuracy and the facility with which they can be controlled. The most significant factor to which these limits may be attributable may be changes in the plasticity of neuronal activities and functions induced by the use of BMI (Zacksenhouse et al., 2007; Ganguly et al., 2011). In most BMI experiments based on the decoding approach, conversion of neuronal signals is aided by appropriate transform algorithms to generate the adequate control parameters. However, the conversion parameters obtained for one set of trials provided increasingly poor predictions of future responses, indicating a source of drift over tens of minutes. Therefore, accurate device control under BMI conditions depends significantly on the degree to which the neuronal activity can be volitionally modulated even for experiments not based on neuronal operant conditioning. Research on such volitional modulation of neuronal activity can be conducted by investigating neuronal operant conditioning, which contributes to realization of a higher performing BMI. The other significant factor affecting the limited performance of the current BMIs may be the bias on firing rate or amplitude of neuronal activity as the source signals. As emphasized in the present paper, synchronous and oscillatory activities have the potential to be neuronal signals constantly representing valid information in the brain. Research for volitional modulation of neuronal synchrony and oscillation by neuronal operant conditioning may contribute much to development of a higher performing BMI.

The issue of neuron selectivity and multipotentiality in the study of neuronal operant conditioning is also a significant issue in BMI studies. Many BMI studies first obtain an optimal basis for brain control by recording the neural activity associated with real limb movement from precentral motor cortex and deriving appropriate transform algorithms (Chapin et al., 1999; Taylor et al., 2002; Carmena et al., 2003; Hochberg et al., 2006; Koike et al., 2006; Choi et al., 2009). However, several other studies demonstrated the ability to extract movement predictions from neurons in postcentral as well as precentral cortical areas (Wessberg et al., 2000; Carmena et al., 2003). Precentral motor neurons could provide the accurate predictions of force and displacement even in small numbers (Koike et al., 2006; Choi et al., 2009), but many neurons from other areas also had potential to provide significant predictions (Wessberg et al., 2000; Carmena et al., 2003). The prediction accuracy increased with the number of neurons included, even when the included neurons were randomly selected and not related to motor movement in nature (Wessberg et al., 2000; Carmena et al., 2003).

Neuronal plasticity, which is inherent in BMI experiments, is not always an obstacle but can be actively applied to induce changes in neuronal connections for functional compensation. For example, Mavoori et al. (2005) investigated the operation of a small computer chip in conjunction with wire electrodes implanted in monkey motor cortex. This “neurochip”, useful for invasive BMI, can convert firings of a cortical neuron not to control signals for external devices but to stimuli directly delivered to other neurons and regions to appropriately modify the neural activity in these regions. Jackson et al. (2006) configured the neurochip as action potentials recorded at one site triggered synchronous stimulation at the neighboring site in the monkey motor cortex. Continuous operation for a day or more resulted in long-term changes in the output effects evoked from the recording site and the changes remained stable for more than a week of testing after the conditioning had terminated. Such conditioning effects were related to time-dependent plasticity and obtained only when the delays between neuronal firings and stimuli were less than 50 ms, indicating that firing synchrony could be involved as an effective factor in such plastic changes.

Finally, we introduce the recent findings that learning to operate BMI induces synchronous and oscillatory activity in other brain regions related to specific functions. Koralek et al. (2012) investigated the role of corticostriatal plasticity, usually involved in learning physical skills, in abstract skill learning using BMI. The authors trained rats to learn to control the pitch of an auditory cursor to reach one of two targets by modulating firing activity in the motor cortex independently of physical movement. During the learning of BMI, alteration of activity was observed in striatal neurons, with more neurons modulating activity in relation to the progress in learning to reach the targets. Concurrently, strong correlations, reflected in oscillatory coupling, between the neuronal activity in the motor cortex and the striatum emerged. This suggests that corticostriatal plasticity and oscillatory interaction underlying physical skill learning is also necessary for abstract skill learning using BMI and that neuroprosthetic movements capitalize on the neural circuitry involved in natural motor learning. Most recently, Koralek et al. (2013) also reported that coherence of activity between motor cortex and striatum during learning of the BMI task is selectively increased in neurons controlling behavioral output relative to adjacent neurons. The temporal offset of these oscillatory interactions aligned closely with corticostriatal conduction delays, demonstrating highly precise timing. Firings from either region were followed by a consistent phase in the other region, suggesting that network feedback reinforces the coherent activity. The authors conclude that temporally precise coherence develops during learning specifically in motor output-related neuronal populations and oscillatory activity serves to synchronize widespread brain networks to produce adequate behavior. This confirms that selective temporal coordination between neurons leading to development of cell assemblies is fundamental in learning to control behavior. Koralek et al. (2012, 2013) reliably indicate that research using BMI can be research of system neuroscience and can provide significant data to reveal normal brain functions and their mechanisms.

In conclusion, research into neuronal operant conditioning, neurorehabilitation, BMI, and system neuroscience will produce findings applicable to all these interrelated fields, and synchrony and oscillation of neuronal activity can be a common key bridge interrelating these disciplines.

## Conflict of interest statement

The authors declare that the research was conducted in the absence of any commercial or financial relationships that could be construed as a potential conflict of interest.
